# An appraisal of the role of asymmetric dimethylarginine on endothelial impairment in preeclampsia comorbid with HIV infection

**DOI:** 10.3389/fmed.2026.1711306

**Published:** 2026-05-07

**Authors:** Mbuso Herald Mthembu, Samukelisiwe Sibiya, Jagidesa Moodley, Nompumelelo Prudence Mkhwanazi, Thajasvarie Naicker

**Affiliations:** 1 Department of Obstetrics and Gynaecology, Nelson R. Mandela School of Medicine, School of Medicine, University of KwaZulu-Natal, Durban, South Africa; 2 HIV Pathogenesis Programme, Doris Duke Medical Research Institute, School of Medicine, College of Health Sciences, University of KwaZulu-Natal, Durban, South Africa; 3 Independent Scholar (retiree), KwaZulu-Natal, South Africa

**Keywords:** ADMA, ART, endothelial impairment, HAART, HIV, HIV proteins, preeclampsia

## Abstract

Asymmetric dimethylarginine (ADMA) is an endogenous eNOS competitive inhibitor that plays a critical role in regulating the bioavailability and homeostasis of nitric oxide (NO) and vascularity. The conditions of both preeclampsia (PE) and HIV infection have their endothelial impairment as their basis, and their prevalence is very high in sub-Saharan Africa, as well as their frequent comorbidity during pregnancy. Elevated ADMA amount suppresses the NO synthesis, which stimulates vasoconstriction, oxidative stress, and inflammation in the vascular wall, which are pathogenic events in PE and cardiovascular risk in HIV. While ADMA has been examined in PE and HIV research separately, its role in women presenting with both conditions during pregnancy is not well understood. Therefore, we conducted a critical review to determine whether ADMA acts as a mechanistic bridge between the involvement of HIV-mediated immune activation, oxidative stress, and the antiretroviral therapy (ART)-induced vascular perturbations, and the endothelial dysfunction that is typical of PE. We evaluated the PRMT-DDAH1-ADMA eNOS axis, outlining the growing evidence that DDAH1 and DDAH2 are the main metabolic enzymes of ADMA, and that ADMA activity is extremely vulnerable to oxidative inactivation. We compared evidence, based on PE-only, HIV-only, and HIV+PE cohorts, and combined the current primary clinical research, which revealed high ADMA levels, poor L-arginine/ADMA ratios, and DDAH gene polymorphism in HIV-associated PE in women. These findings suggest that inflammatory signaling and reactive oxygen species are associated with HIV infection and can potentially inhibit the activity of DDAH1 and increase PRMT-mediated methylation, contributing to systematic accumulation of ADMA and a deficiency of NO during pregnancy. We also investigated how the proteins of HIV infection (gp120, Tat, and Nef) mediate the increase in endothelial activity and oxidative stress, and explained how these proteins may have an indirect effect on the regulation of ADMA via inflammatory and redox-sensitive pathways. Even though the initiation of ART has been observed to be correlated with the decrease in circulating levels of ADMA in non-pregnancy HIV cohorts, it is not clear what the clinical effect of this decrease in pregnancy is, namely, immune reconstitution, angiogenic imbalance, and PE susceptibility. Thus, filling in mechanistic, genetic, and clinical evidence, our study determines the gaps in learning about the ADMA–HIV–PE axis and measures whether the modulation of the L-arginine/ADMA ratio or DDAH1 activity is a plausible diagnostic or treatment option. We present that an understanding of this pathway is key to the development of biomarkers and in the development of focused, situation-specific interventions, where resources are scarce, and the burden of disease is increasing.

## 1 Introduction

Impairment of the endothelium, found in several inflammatory and cardiovascular diseases, is an important contributor to the pathogenesis of preeclampsia (PE) (1). It is estimated by the World Health Organization (WHO) that diseases associated with pregnancy-related hypertension cause approximately 10% of all maternal deaths, with the highest prevalence affecting Asia and Africa (2). Hypertensive disorders in pregnancy (HDP) are the most common cause of maternal deaths in South Africa (SA), contributing to 16.9% of all maternal deaths; the majority of these deaths result from PE (3). The mortality rate of HDP among mothers has reduced by 18% in the last three triennia (4). Pregnancy-related infections are not the only infectious diseases that led to 27.1% of maternal deaths in 2020 (5). In 2020, SARS-CoV-2 pneumonia exacerbated the number of maternal deaths due to the COVID-19 pandemic (6). PE is estimated to impact about 5%−10% of pregnancies in the world, and the additional levels of maternal and newborn mortality and disease, especially in sub-Saharan Africa (7). This issue is exacerbated by the high level of HIV infections in the region. The number of women living with HIV in sub-Saharan Africa is estimated to be 20.8 million, which constitutes more than 58% of the total HIV-positive population in the world (8).

The transmission of HIV infection has been linked to endothelial dysfunction based on the issues of long-term immune stimulation, elevated levels of oxidative stress, and the interruption of nitric oxide (NO) bioavailability (9, 10). Asymmetric dimethylarginine (ADMA) has a dual mechanism since it is a natural nitric oxide synthase inhibitor (eNOS) and an important mediator of endothelial dysfunction (11). PE and HIV-infected individuals are both reported to develop an increase in plasma ADMA, indicating that both conditions may have a pathophysiology relationship that may aggravate the condition when they both occur concomitantly (12, 13). ADMA inhibits the synthesis of nitric oxide synthases competitively and decreases the bioavailability of nitric oxide, which connects the disturbances linked to the signaling of nitric oxide with the endothelial dysfunction (11, 14). A review of the literature in this line show that the dysfunctional interactions between ADMA and immunomodulators in the blood, as well as vascular risk, are elevated in HIV-positive patients, indicating that ADMA is a marker of endothelial dysfunction in HIV-positive individuals (15, 16), suggesting that these illnesses may share a pathophysiological relationship that could worsen vascular dysfunction when they co-occur (12, 13).

Despite this overlap, existing research has yet to establish the specific role of ADMA in endothelial impairment among women who develop PE and those living with HIV (17, 18). As inhibitors of NO synthesis and a biomarker of endothelial health, both diseases are issues of public health significance that might have close links, and it is important to understand the crossroads of both. To this end, the purpose of the current study is to determine whether ADMA–DDAH–NOS signaling axis dysregulation is a mechanistic association between immune activation in HIV and antiretroviral therapy and vascular perturbations and preeclampsia-associated endothelial dysfunction. In answering this question, the evidence has been denoted on three levels, namely: (i) studies exploring ADMA in preeclampsia in isolation, (ii) studies exploring ADMA and endothelial dysfunction in HIV infection, and (iii) recent clinical and genetic trials of ADMA pathways in women with HIV-related preeclampsia.

Electronic databases, such as PubMed, Web of Science, Scopus, Google Scholar, ScienceDirect, Wiley Online Library, and SpringerLink, were searched for relevant literature. Keywords that were combined in the search strategy included: asymmetric dimethylarginine, ADMA, preeclampsia, HIV, endothelial dysfunction, and nitric oxide. Original clinical studies, mechanistic research, and recent reviews on ADMA metabolism, nitric oxide signaling, and endothelial dysfunction during pregnancy and in HIV infection were prioritized. Articles published since 1990, especially recent research studies on the ADMA-2/DDAH-2/NOS axis and vascular dysfunction, were included.

## 2 Endothelial biology and impairment

### 2.1 Endothelial biology

The vascular endothelium lines the heart, lymphatic vessels, and blood vessels with a single layer of squamous epithelial cells. It is considered the largest endocrine organ in the body (19, 20). The endothelium invests the entire vascular system and plays a key role in maintaining vascular balance (21, 22). Its ability to control vascular tone first showed its importance. A healthy endothelium releases molecules that help maintain the stability of the vascular wall. It also responds to circulating substances, such as bradykinin and thrombin, modulating their effects (23, 24). Over time, additional roles have been found. The endothelium regulates inflammation and coagulation, promoting and blocking vascular growth factors (25, 26). Endothelial cells adjust their actions to restore balance in response to various challenges (27, 28). They release substances that widen blood vessels. Endothelium also increases constriction by making vasoconstrictors like endothelin and other prostanoids, and by converting angiotensin I to angiotensin II (29, 30). The endothelium also helps keep blood vessels relaxed by affecting smooth muscle cells through a pathway that does not use NO. This action increases potassium flow and alters the function of these muscle cells (31–33). In this way, endothelial cells form a complex tissue. They act as a controlled barrier between blood and tissue (34–36). Due to the key role of NO signaling in endothelial homeostasis, endogenous NO synthase inhibitors, including ADMA, are becoming important regulators of endothelial behavior and vascular health (14, 37).

### 2.2 Pathophysiology of endothelial impairment

**Figure 1:**
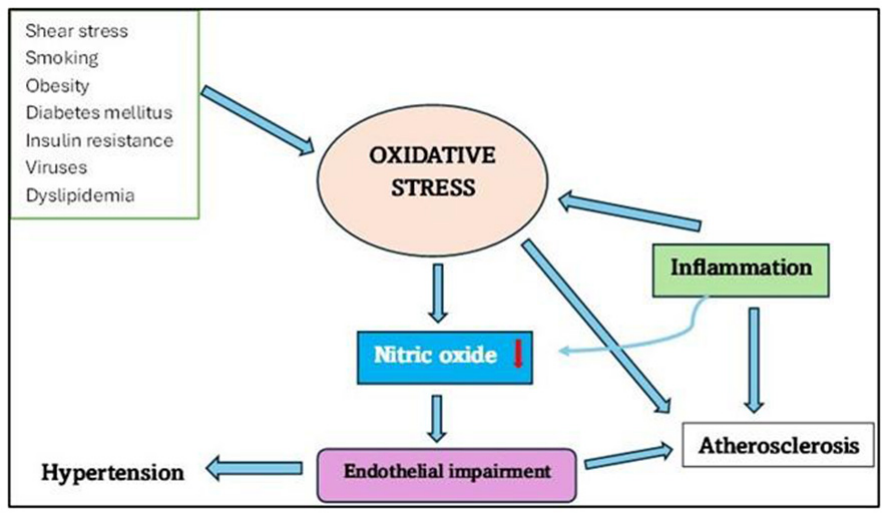
Schematic diagram illustrating the link between inflammation, chronic diseases, and Oxidative stress. They dysregulate nitric oxide thus causing endothelial impairment in various conditions. Identifying endothelial damage early is crucial in averting atherosclerosis and cardiovascular disease, as this dysfunction could represent a preliminary, reversible stage in the development of atherosclerosis (44).

Endothelial impairment is a multifactorial disorder that affects blood vessels through the destruction of a thin layer (38). Reduced NO availability signals this problem (39, 40). NO keeps blood vessels healthy by widening them and preventing the formation of clots. In endothelial impairment, oxidative stress and inflammation lower NO (41, 42, 226). Endogenous NOS inhibitors, especially ADMA, can also accumulate and increase the inhibition of nitric oxide and result in endothelial dysfunction (11, 14). The occurrence of oxidative stress destroys the cell when the ratio of oxidants exceeds the antioxidants or when the body is incapable of repairing (41). Conditions such as hyperglycemia, insulin resistance, abnormal blood fats, inflammation, and smoking all promote oxidative stress and reduce NO (41) (Figure 1). Cellular damage is caused by reactive oxygen species (ROS). They are normally eliminated by the body, but oxidative stress over prolongs defenses (41). Free radicals disrupt the balance of NO, making the endothelium too permeable and allowing toxins and certain cells to cross from the blood into nearby tissues (20, 43). The liver produces proteins, such as C-reactive protein (CRP), which play a role in inflammation (20). Inflammation also reduces NO, and CRP reduces eNOS activity (20).

Occasionally, NO reacts with superoxide to form peroxynitrite, a very harmful substance that breaks down the eNOS cofactor tetrahydrobiopterin (BH4) (45). As a result, eNOS stops making NO and instead produces more ROS (41, 46). Oxidative stress is closely linked to inflammation in blood vessels (46, 47) and can also lead to cell death, particularly programmed cell death (apoptosis) (41). In short, endothelial impairment upsets the balance of NO, harms blood vessel function, and increases cardiovascular risks. Vascular endothelial impairment characterizes PE, particularly glomerular endotheliosis (48–52), which raises significant cardiovascular risks during and after pregnancy (53).

### 2.3 Endothelial impairment in preeclampsia

PE features poor blood flow to the placenta and excessive inflammatory mediators, which damage vascular endothelial cells and cause functional problems that contribute to high blood pressure (54). Endothelial cells (ECs) play crucial roles by sensing blood contents and acting as barriers to prevent the movement of water, ions, proteins, and cells from the blood into the vessel wall, roles that PE disrupts (55). As a result, new blood vessel growth and widening of existing vessels in response to stress or low oxygen levels insufficiently occur (55, 56). In PE, ECs become less sensitive to vasoconstrictors and display poor signaling, despite increased vasodilator production (57). Lower circulating cyclic nucleotide levels suggest that EC coupling increases, potentially impairing calcium signaling. In PE, ECs exhibit reduced calcium signaling, resulting in lower NO production (55, 57). Elevated ADMA levels reported in preeclamptic pregnancies may further suppress NO synthesis and exacerbate endothelial dysfunction (58, 59).

A common result of endothelial impairment in PE is a prothrombotic state, caused by the release of procoagulant factors from damaged ECs and by an increased number of white blood cells moving to the injured area (60). Endothelial impairment and higher resistance in blood vessels are key parts of PE in mothers (61). Abnormal placental development, reduced blood flow, and the release of placental products all damage the vascular endothelium (52, 62, 63). Clinical assays show that markers of endothelial activation and flow-mediated dilation (FMD), which measures endothelial function, are reduced in people with PE (49, 64, 65). Studies have found higher levels of lipid peroxides, interleukin-8, and the antioxidant SOD in ECs from women with PE (66). Recent studies have found that women with PE have higher levels of IL-8 and lipid peroxides in their blood, which may affect how easily substances pass through the endothelium (67, 68). This increased permeability is also linked to changes in the distribution of proteins that connect endothelial cells (35, 69).

## 3 Pathophysiology of preeclampsia

PE is a human pregnancy-specific disorder and a major cause of maternal and perinatal morbidity and mortality (70, 238). PE manifests clinically after 20 weeks of gestation and is characterized by significant proteinuria defined as ≥2+ by urinary dipstick, ≥0.3 g/day by 24-h urine collection, or ≥30 mg/mmol by spot urine protein: creatinine ratio with maternal symptoms including headache (new, persistent or unusual), herein hypertension defined as a systolic BP (sBP) ≥140 mmHg and/or diastolic (dBP) ≥90 mm Hg, based on an average of at least two measurements, visual disturbances, persistent right upper quadrant or epigastric pain, severe nausea or vomiting, chest pain or dyspnea and maternal signs including eclampsia, pulmonary oedema and placental abruption (71). PE may be classified by gestational age and severity. Based on the onset of PE, it may be differentiated into early-onset preeclampsia (EOPE) and late-onset preeclampsia (LOPE). EOPE occurs before 34 weeks of gestation, characterized by abnormal placentation, which leads to endothelial dysfunction, hypoxia, oxidative stress, and severe angiogenic imbalance. In contrast, LOPE occurs at or after 34 weeks and can lead to maternal cardiovascular risk, endothelial dysfunction, and severe maternal organ damage such as the liver, brain, and or kidneys (72–74).

The pathogenesis of PE is believed to originate from placental hypoxia, oxidative stress, a reduction in uterine natural killer cells, an exaggerated inflammatory response, and angiogenic imbalance resulting from defective spiral artery transformation, which leads to the release of maternal factors that cause widespread endothelial injury (75–77). PE is also recognized as a predisposing factor to the development of cardio-metabolic conditions such as hypertension and diabetes later in life (76, 78).

## 4 Human immunodeficiency virus infection comorbid with preeclampsia

The opposing response of PE neutralizes the exaggerated inflammatory response; therefore, HIV infection can prevent the development of PE (79). No conclusive information is available concerning the synergy of HIV infection of PE. Nevertheless, a report showed that rates of PE were much lower among the population living with HIV (PLWHIV) than in HIV-negative pregnant women (80). Several studies have shown a reduced incidence or no incidence in prevalence of PE predisposition between HIV-uninfected and HIV-infected women on highly active antiretroviral therapy (HAART) (81, 82). A case-control study found that untreated HIV infection has a protective effect against the onset of PE, but it was not reported in women under antiretroviral therapy (83). Equally, according to a recent observational study carried out on a cohort of 4,078 pregnant women in Zambia, it was found that among 4,078 pregnant women, PE was identified in 186 individuals (4.6%), although the incidence of PE was lower among women living with HIV (2.7%) than in those who were women without HIV (5.8%) (84).

One of the studies revealed a higher incidence of PE among HIV-infected women than non-infected women, which was due to the immune reconstitution effect of HAART (85). Other research studies that compared HIV-infected women on receipt of HAART than HIV-uninfected women established a greater vulnerability of developing PE among HIV-infected women on receipt of HAART (86, 87). Nevertheless, a study showed that the risk in HIV-infected women on HAART was at lower rates (83) compared to other studies (88, 89), which were also statistically significant (83). This putative protective effect of HIV infection on preeclampsia is thus not always convincingly evidenced in various studies, thus a great heterogeneity in the literature and a likely dependence on immunological, inflammatory, and endothelial processes in modifying disease susceptibility in specific populations.

Some of the biomarkers related to endothelial dysfunction have been examined with regard to HIV-associated preeclampsia. These include prostacyclin (PGI2), intercellular adhesion molecule-1 (ICAM-1), vascular cell adhesion molecule-1 (VCAM-1), and asymmetric dimethylarginine (ADMA), and each of them has significant functions in the process of endothelial homeostasis and in vascular inflammation. The hypertensive pregnancy disorders have been reported to be altered with changes in these mediators and could be even enhanced by the HIV-related immune activation and antiretroviral therapy. ADMA is one such biomarker that has drawn special attention due to its direct inhibition of endothelial nitric oxide synthase and interference with nitric oxide bioavailability, which is a major predictor of endothelial function (11, 14). Higher ADMA levels have been seen in preeclampsia as well as in infection with HIV, implying that an imbalanced ADMA–nitric oxide pathway could be a possible putative mechanism of interrelation between the two. Therefore, the role of ADMA in the coexistence of HIV infection and preeclampsia can be of great value in the mechanisms that have led to endothelial impairment in this comorbid state.

## 5 ADMA biology and vascular regulation

**Figure 2:**
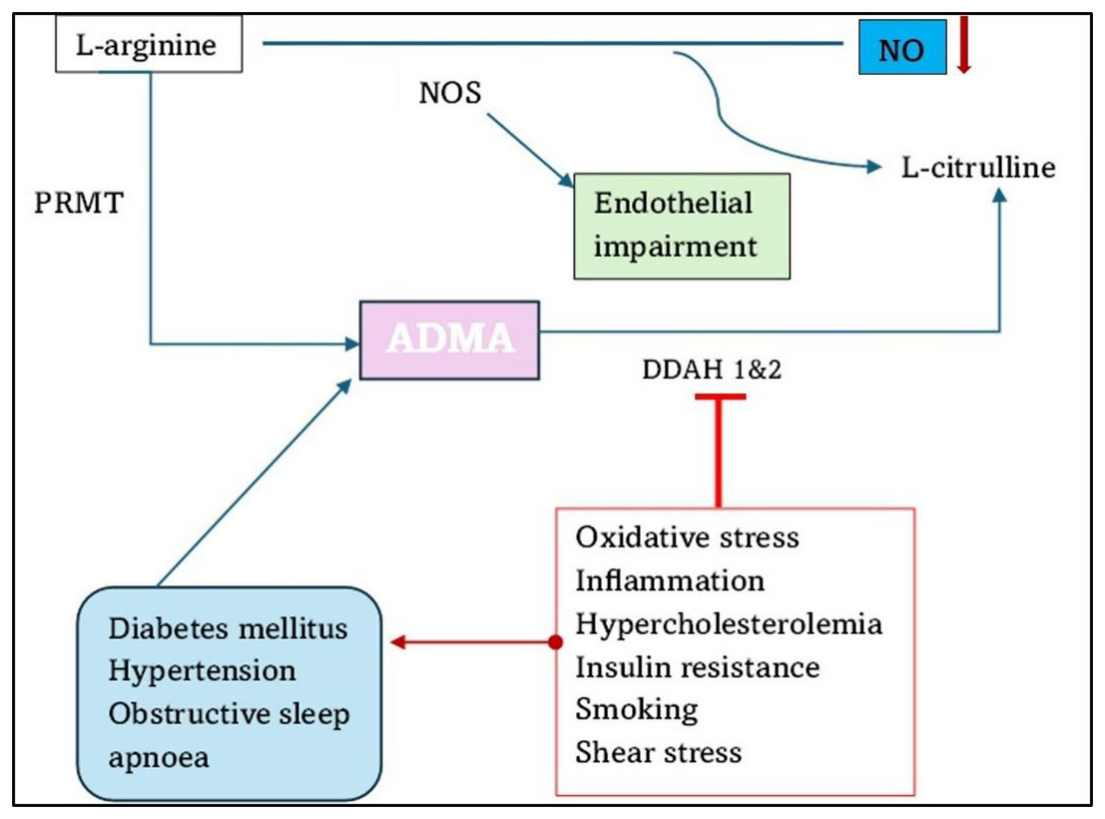
Origin and fate of asymmetric dimethylarginine (ADMA). ADMA derives from methylation of arginine residues in proteins. The reaction is catalyzed by protein arginine N-methyltransferases (PRMT). Hydrolysis of the methylated proteins releases ADMA, which competitively inhibits nitric oxide synthase (NOS) thus affecting NO production. ADMA is hydrolyzed by the enzyme DDAH to form L-citrulline, asymmetric dimethylarginine; DDAH, dimethylarginine dimethylaminohydrolase; PRMT, protein arginine methyltransferase; NO, nitric oxide; NOS, nitric oxide synthase (adapted from (14)).

ADMA, a methylated product of L-arginine, is a natural inhibitor of endothelial nitric oxide synthase (eNOS) (90, 91). It is produced by the action of protein arginine methyltransferase (PRMTs); hence, it may contribute to endothelial dysfunction (14, 90, 92, 93). ADMA is dynamically metabolized by dimethylarginine dimethylaminohydrolase (DDAH), which is expressed in two isoforms: DDAH1 and DDAH2. DDAH 1 is extensively expressed, especially in the liver and proximal convoluted tubules of the kidney, which are sites of NOS expression (94, 95). DDAH 2 is expressed at relatively high levels in fetal tissues; however, in adults, its concentration decreases, and the sites of expression become more selective (95). Recent biochemical and genetic evidence showed that DDAH1 is the major enzyme that metabolizes ADMA *in vivo*, whereas DDAH2 seems to have a more restricted or regulatory role in vascular tissues. With these results, the ADMA–DDAH1–NOS signaling axis is relevant to the maintenance of the bioavailability of nitric oxide as well as the homeostasis of endothelial cells. Nevertheless, beneficial effects on endothelial impairment were observed in patients at risk of vascular disease with increased ADMA, such as hypercholesterolemia, but not in hypertension or diabetes (96). Conjecturally, numerous risk factors such as hypertension (97), hypercholesterolemia (37, 232), obesity (98, 232), diabetes mellitus (99), obstructive sleep apnea (100), and vascular inflammation (101) may mediate their detrimental effects on the vascular wall through the impairment of the endothelial L-arginine/NO pathway (Figure 2). Nitric oxide is a potent, highly reactive signaling molecule that maintains endothelial integrity by regulating vasodilation, adhesion of leukocytes to ECs, angiogenesis, and regulation of platelet function (102–104). During pregnancy, the concentration of endogenous eNOS inhibitors is significantly lower (105). There is a dysregulation of ADMA in PE; however, no work is available on the synergy of HIV infection comorbid with PE (58, 106, 107).

### 5.1 Asymmetric dimethylarginine in preeclampsia

Of note, in PE, there are significantly higher levels of ADMA than in normotensive, gestational-age–matched women (106, 108–111). Maternal ADMA concentrations were elevated at mid- and late pregnancy in women who later developed PE (59). Increased ADMA concentration contributes to the development of PE in early pregnancy, and consequently poor placentation notably, a higher concentration of ADMA is observed in early-onset PE, which may explain the link between disease severity and the timing of clinical manifestation (108, 112) (Table 1). Several endothelial biomarkers have undergone research on the pathogenesis of preeclampsia, which are soluble fms-like tyrosine kinase-1 (sFlt-1), vascular endothelial growth factor (VEGF), placental growth factor (PlGF), intercellular adhesion molecule-1 (ICAM-1), vascular cell adhesion molecule-1 (VCAM-1), endothelin-1, and prostacyclin. All these mediators are indications of angiogenic imbalance, inflammatory responses, and dysfunction of the endothelium in hypertensive pregnancy disorders (54, 76, 113).

**Table 1:** Studies reporting the effect of ADMA in preeclampsia.

References	Plasma/ serum	ADMA effect in preeclampsia
(106)	Plasma	↑
(107)	Plasma	↑
(127)	Plasma	↑Mild and severe PE
(216)	Plasma	No significant difference between PE and normotensive
(59)	Plasma	↑
(109)	Plasma	No significant difference between PE and normotensive
(108)	Serum	↑
(110)	Plasma	↑
(111)	Serum and placenta	↑
(217)	Serum	↑
(218)	Serum	↑
(219)	Blood	↑

### 5.2 Asymmetric dimethylarginine in HIV infection

ADMA is a natural inhibitor of NOS that influences endothelial-dependent vascular relaxation and host defense against infection (11). *In vitro* and *in vivo* investigations indicate that the accumulation and ADMA production are associated with the inflammatory and immune activation cascades (114). HIV-infected patients have an increased plasma ADMA and SDMA. Methylated arginine species are associated with immunological activation in HIV infection (15). The HIV-1-encoded trans-activator (Tat) and the HIV-1 nucleocapsid protein are also targets for PRMT6, whereby the protein asymmetrically methylates arginine residues (115–117) to maintain optimal HIV-1 infectivity.

Oxidative and inflammatory mechanisms have also been reported to be involved in endothelial injury due to HIV-1 viral proteins. *In vitro* and *in vivo* experimental studies have demonstrated that HIV-1 gp120 and Tat will induce endothelial reactive oxygen species, inflammatory signaling, and endothelial dysfunction, which indicates oxidative stress as a mediator of vascular damage (83, 84, 118). Viral proteins can induce oxidative stress, which can inhibit dimethylarginine DDAH activity, which will decrease the degradation of ADMA, thus favoring the accumulation of the same within the vascular endothelium (119, 120).

Also, inflammatory signaling that accompanies HIV can increase the activity of protein arginine methyltransferases (PRMTs), which carry out the process of methylating arginine residues in proteins. In the process of subsequent proteolysis of such methylated proteins, the ADMA is lost into the circulation, which adds to high levels of ADMA in the case of HIV infection (121–123). The combination of these mechanisms provides a biological cause for these elevated levels of ADMA in people with HIV and indicates the role of viral proteins, oxidative stress, and ADMA metabolism in interaction.

The levels of ADMA are higher in younger patients who have higher levels of baseline C-reactive protein and D-dimer (124) or neopterin, which are predictors of a higher risk of cardiovascular events independent of other risk factors (125, 126). These findings aid in enhancing the comprehension of the endothelial harm and malfunction associated with HIV infection and recommend that increased inflammatory and coagulation cascades condition prematurely inclusive vascular hampering and illness in patients with HIV infection (124).

### 5.3 Asymmetric dimethylarginine in the comorbidity of HIV and PE

It has been found that PE has higher maternal plasma ADMA levels (58, 107). Moreover, it is not a secret that high ADMA levels tend to be a setup for the emergence of PE (12, 58, 106, 107, 127). Immune activation is associated with increased ADMA levels in the plasma of HIV-infected patients (128). The augmented ADMA of HIV and decreased L-arginine of PE may influence the NOS action amidst the setting of PE, which is directly related to HIV (16, 129).

The role of ADMA in preeclampsia related to HIV has recently been studied. A study of the ADMA and prostacyclin roles in endothelial dysfunction in parity of HIV-associated preeclampsia was carried out by our research team and registered high levels of ADMA in the HIV-negative PE group as opposed to the normotensive HIV-negative group (17). We also found more ADMA in PE than in normotensive pregnancy, regardless of HIV, and indicated a positive correlation between higher levels of ADMA and development of preeclampsia (17).

**Figure 3:**
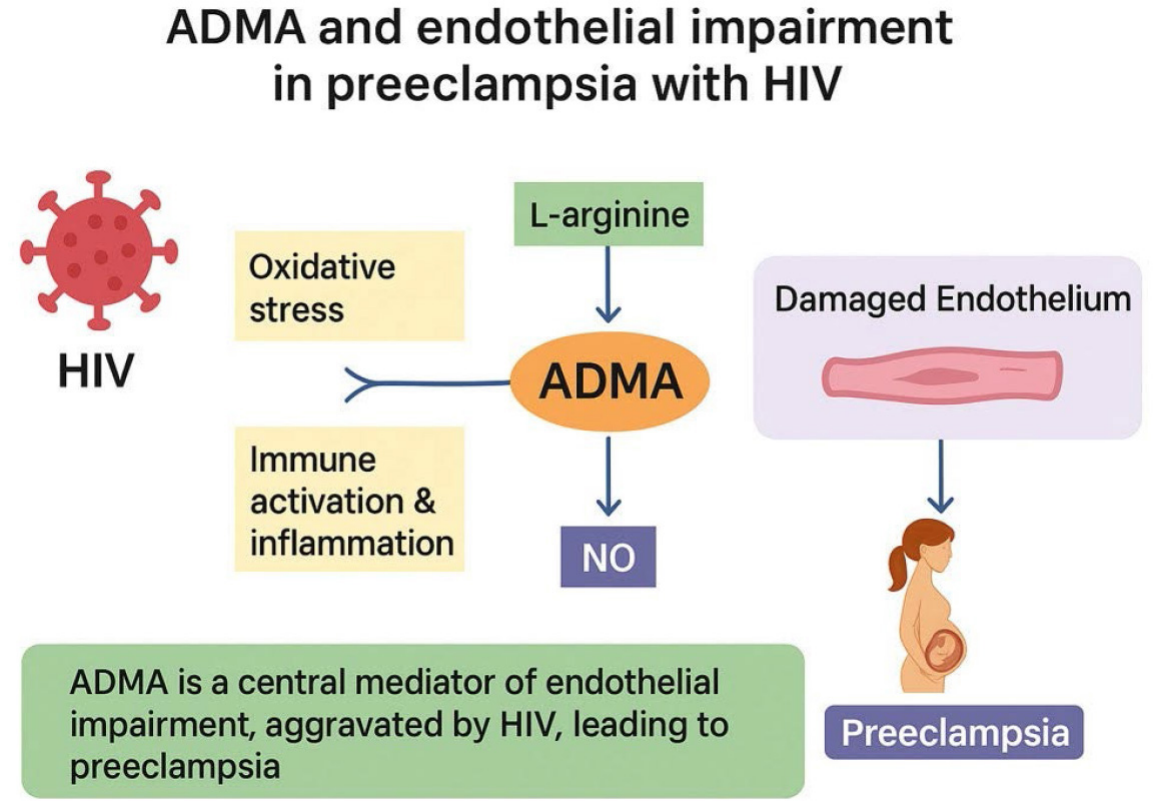
Schematic diagrams showing the ADMA–HIV–PE axis.

Moreover, genetic studies examining dimethylarginine dimethylaminohydrolase polymorphs have shown that there is a relationship between the variants of DDAH and the susceptibility to HIV-associated preeclampsia in women of African descent (18). ART reduces the ADMA and SDMA levels in the plasma (15, 128, 130). Nevertheless, while new evidence indicates that ADMA dysregulation may be linked to endothelial dysfunction in HIV-related preeclampsia, data on this topic are scarce, and more research is needed to shed more light on the mechanistic interplay between HIV infection, ADMA metabolism, and PE development. All these mechanisms pose the possibility of an integrated ADMA–HIV–preeclampsia axis of immune activation, oxidative stress, and endothelial dysfunction. Figure 3 is a schematic diagram of this proposed ADMA–HIV–PE axis.

### 5.4 ADMA and antiretroviral therapy

The initiation of ART decreases ADMA levels compared to an individual with ARV naive; however, it is unclear whether the decline in ADMA levels affects the risk of disease (15, 124, 130). A previous study in individuals with ART naïve demonstrated significant correlations between the immune activation marker neopterin and dimethylarginine levels (15). Also, patients with ART naïve display an association between inflammatory cytokines and endothelial activation markers (131). A study demonstrates that ART effectively slows endothelial activation, as indicated by declining ADMA levels (128). In addition, decreasing ADMA concentration may be associated with ART-mediated downregulation of immune activation cascades (128).

The WHO guidelines for the management of pregnant women living with HIV recommend initiation of HAART early in pregnancy and increasing the eligibility criteria from a CD4 cell count threshold of ≤ 200 cells/μl to ≤ 350 cells/μl or WHO clinical staging 3 or 4, regardless of gestational age (132, 133). Since 2016, South Africa has employed a “test and treat all” strategy, providing ART to all people living with HIV, irrespective of their CD4 cell count (134). However, there is little evidence for the safety of HAART during pregnancy as it has been correlated with adverse outcomes such as prematurity, low birth weight (135), and an increased rate of gestational diabetes (86). HAART was also reported to increase the risk of severe PE development (136). Before the development of HAART, certain nucleoside/nucleotide reverse transcriptase inhibitors (NRTIs) were used to control HIV infection.

The development of NRTIs, viz., azidothymidine, tenofovir disoproxil fumarate, and lamivudine, has successfully curbed HIV infection into a controllable medical entity (137–139). NRTIs lead to the dysregulation of EC propagation and migration (140). This study further elucidated that NRTIs induce mitochondrial oxidative stress, which encumbers the activation of endothelial receptor tyrosine kinase signals and VEGFR-2 pathways in vascular ECs (140). A mechanical investigation carried out in 2007 by Jiang et al. (141) corroborates these findings. Mitochondrial DNA depletion increases oxidative stress and apoptosis in the placenta of women receiving zidovudine-containing ART, which implicates mitochondrial failure that would promote PE development and resultant adverse perinatal outcomes (142). The disturbance of normal angiogenesis and lymphangiogenesis resulting from the effects of NRTIs may influence the development of PE (140, 142). Since ECs have angiogenic capabilities as one of their basic functions, this characteristic is an indicator of NRTIs' interference with angiogenesis. Therefore, the ARTs used to suppress HIV could be potent anti-angiogenic molecules. The long-term or continued use of NRTIs significantly aggravates endothelial impairment, notably in the intima-media remodeling of the aorta and carotid artery, as demonstrated in a mouse model (143–145).

Protease inhibitors (PIs) impede HIV aspartyl protease, resulting in immune reconstitution, which may predispose women to PE development (146). It was reported that the two commonly prescribed ARTs, namely AZT and Indinavir, can cause direct endothelial impairment as shown by decreased endothelium-dependent vasodilation and increased plasma levels of endothelin-1 (147). The toxic effects resulting from the administration of PIs are a consequence of dysregulation of glucose and lipid metabolism, which often leads to energetic failure and can ultimately result in apoptosis (148, 149). Endothelin-1 was reported to be dramatically increased in ECs treated *in vitro* with ART, resulting in increased vasoconstriction (150). It has also been reported that ART initiation can lower the estimations of ADMA by inhibiting systemic inflammation and stimulation of the immune system in individuals with HIV (84, 119). Nevertheless, even with a potential decrease in ADMA levels, endothelial dysfunction can continue to take place due to some antiretroviral drugs causing oxidative stress, mitochondrial damage, and disturbances of metabolic processes that separately contribute to the deterioration of the vascular status. These results indicate that the vascular actions of ART are complicated and can include both positive-affirming action of immune activation and negative-affirming action of oxidative endothelial damage.

## 6 HIV proteins as mediators of ADMA-associated endothelial impairment

**Figure 4:**
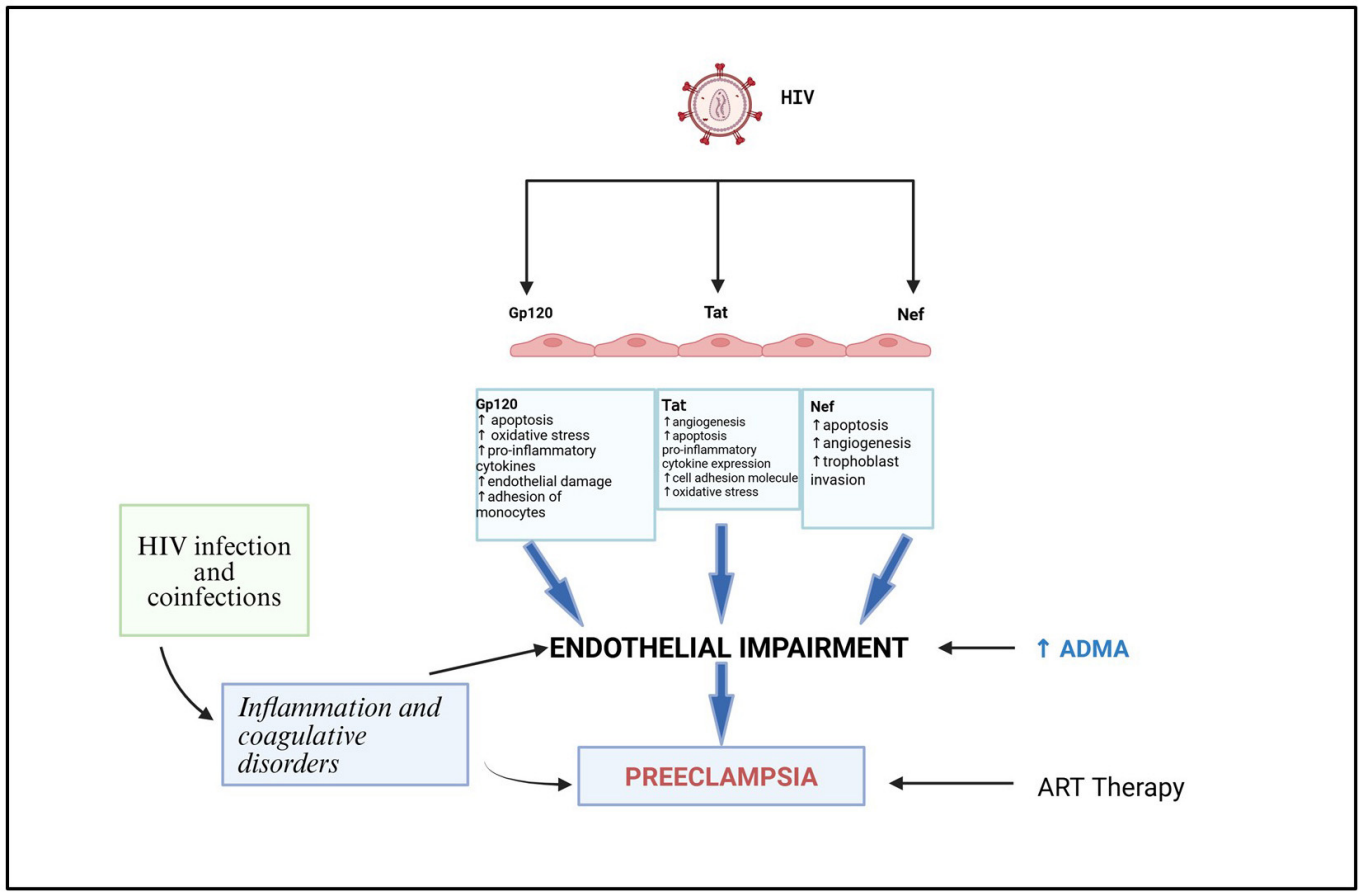
HIV proteins and their role in endothelial impairment. Image adapted from (151).

Untreated HIV infection is associated with endothelial impairment (152). The HIV-1 proteins Tat, gp120, and Nef, together with the proinflammatory cytokine TNF-α, and the ARTs efavirenz and lopinavir, are hypothesized to be primary causal agents of endothelial impairment (151, 153) (Figure 4). These viral proteins facilitate endothelial dysfunction through the same mechanisms that include oxidative stress, inflammatory signaling, and interference with NO bioavailability, which are intimately connected with the accretion of an endogenous nitric oxide competitor, asymmetric dimethylarginine. ADMA is an endogenous competitive inhibitor of endothelial nitric oxide synthase (eNOS), which decreases the nitric oxide bioavailability and leads to endothelial dysfunction (11, 14). High levels of ADMA suppress the amount of NO made available and inhibit vascular dilatation, thus leading to injuries in the endothelium in both HIV infection and hypertensive pregnancy diseases like preeclampsia (11, 14). HIV can enter ECs through various pathways, including CCR3 and CCR4, cluster of differentiation 4 (CD4), or galactosylceramide receptors (154, 155). However, entry of the virus is mediated by gp120 and Tat proteins, as well as the participation of cytokines secreted by HIV-infected mononuclear and adventitial cells. Individual viral proteins affect the endothelium in both specific and overlapping ways. Circulating levels of gp120 have been reported at concentrations ranging from 0.24 to 92 ng/ml and are readily detectable in serum, facilitating the virus's entry into host cells (156, 157). It is possible that viral proteins may affect the ADMA/DDAH/NOS signaling axis via redox-sensitivity pathways. HIV protein-induced increased oxidative stress and inflammatory signaling can block the activity of DDAH and increase ADMA accumulation and decrease nitric oxide bioavailability by promoting PRMT-mediated arginine methylation.

In addition, the virus can infect and decrease endothelial progenitor cells (the circulating colony-forming units, ECs), which are essential for repairing endothelial damage (158). Exposure to gp120 increases expression of intercellular adhesion molecule-1 (ICAM-1), but not E-selectin or vascular cell adhesion protein (VCAM), in human coronary artery, umbilical vein, lung, brain, and dermal microvascular ECs (159). Furthermore, gp120 is responsible for the apoptosis of endothelial cells and the stimulation of macrophages to produce excessive amounts of NO, which directly leads to endothelial damage (154, 231, 237). Interestingly, it has been found that exposure to cigarette smoke and HIV gp120 causes a synergistic increase in EC death (154, 160). Moreover, the HIV Tat protein can activate inflammatory pathways through mononuclear cells, which are known to produce tumor necrosis factor-α (TNF-α) and the pro-inflammatory transcription factor nuclear factor kappa-light-chain-enhancer of activated B cells (NF–κB). Tat protein also elicits the expression of adhesion molecules E-selectin, vascular cell adhesion protein 1 (VCAM-1), and ICAM-1, which alter endothelial permeability (154, 161). Like Tat, gp120 significantly increases the adhesion of monocytes (159, 162) and lymphocytes (163) to the endothelium. These viral proteins trigger inflammatory and oxidative stress pathways that could also lead to further impairment of the activity of DDAH, which takes part in the metabolism of ADMA. A decrease in DDAH activity encourages the buildup of ADMA and consequent suppression of eNOS-regulated production of nitric oxide, hence enhancing endothelial impairment amid HIV infection (14, 83). Other recent biochemical results even point to the fact that DDAH1 and not DDAH2 is really in charge of ADMA metabolism, which also puts the ADMA to DDAH1 to NOS signaling axis into perspective in the context of vascular biology (120).

The effects of other HIV-1 accessory proteins on EC biology require clarity. Adenoviral expression of Vpu upregulates the TNF receptor family molecule CD40, inducing VCAM-1 expression and adhesion of β-lymphoma cells to the endothelium (164). Treatment of ECs with recombinant Nef induces TNF-α production and EC apoptosis (165). Nef communicates with the endothelium and with HIV-1-infected T cells to upregulate viral replication through direct cell–cell contact or the release of endothelial signals. Nef further dysregulates human monocytes and macrophages by activating the downstream MAPK pathway and regulating the production of superoxide from macrophages, which may contribute to vascular dysfunction (166, 167). Nef not only has direct effects on cell signaling but also activates macrophages to facilitate EC foam cells formation (168). Arterial wall foam cells are also common in PE due to acute atherosclerosis, which is a lesion of the uteroplacental spiral arteries. These foam cells are supposed to derive from permeating CD68-positive macrophages and disintegrating smooth muscle cells (169). Recent studies on HIV-associated preeclampsia have detected high levels of ADMA related to endothelial dysfunction in PE regardless of HIV status, which presents ADMA as a mechanistic factor in vascular dysfunction of the comorbid disease (17). Genetic research that analyses dimethylarginine dimethylaminohydrolase polyphorphs and studies have established links between DDAH gene-based phylogenetic variants and vulnerability against HIV-associated preeclampsia in African descent women, which again implicates the maladaptation of the ADMA metabolic pathway in the development of the disease (18). Significantly, even though ART initiation might potentially lower the level of circulating ADMA due to its potential suppressing influence on systemic inflammation, immune activation, a certain number of antiretroviral drugs have the propensity to, by themselves, promote oxidative stress, mitochondrial dysfunction, and metabolic abnormalities that interfere with the endothelial functioning. Hence, as a rehabilitated marker of endothelial health, the ADMA minimization following the initiation of ART may not be invariably closely linked to the acute restoration of all areas of endothelial health.

### 6.1 HIV proteins and preeclampsia

#### 6.1.1 Gp120

The HIV-encoded proteins, Tat, gp120, and Nef are implicated in endothelial injury (170, 171, 229). Glycoprotein 120 is known to induce increased ROS production, causing oxidative stress and impairing endothelial cell function/or indirectly via macrophages/monocytes in contact with the vessel wall (118, 172–174). It is also known that oxidative stress inhibits the activity of DDAH, which leads to an accumulation of ADMA and the inhibition of endothelial nitric oxide synthase (eNOS) (14). This is one of the ways in which gp120-induced oxidative stress plays a role in ADMA-induced nitric oxide dysregulation and endothelial dysfunction (235).

Furthermore, HIV-1 gp120 is also directly involved in the upregulation of pro-inflammatory cytokines, such as interleukin-6 (IL-6) and interleukin-8 (IL-8), in primary ECs (175, 233). It has recently been associated with several systemic inflammatory responses, including within the hypoxic microenvironment of PE (176). IL-8 levels are increased in PE compared with healthy controls, as demonstrated by Sharma et al. (177) in India and Sahin et al. (178) in Turkey. These findings are also corroborated by Sun et al. (68), who demonstrated that women with PE have higher serum and placental expression of IL-8 compared to healthy pregnant controls.

#### 6.1.2 Tat

**Figure 5:**
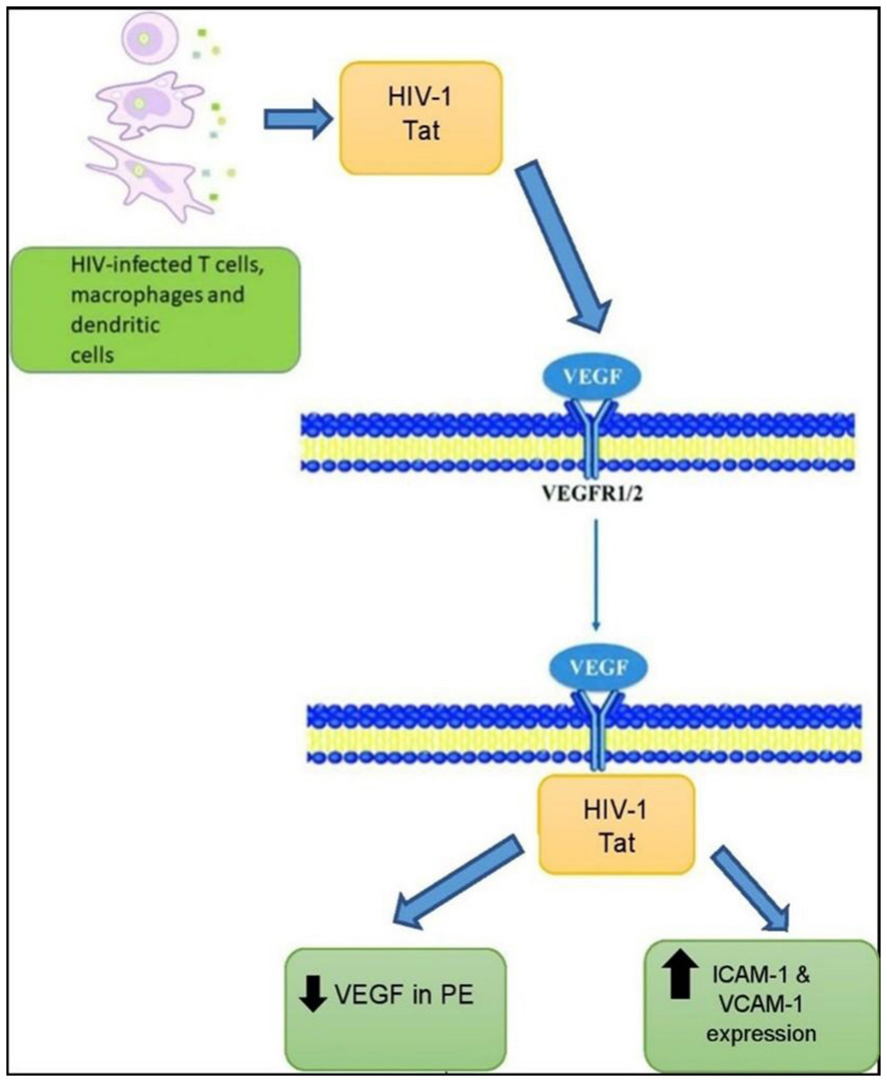
HIV-1 Tat protein in VEGF activity. Tat protein is released from infected cells, T cells, and mimics VEGF activity by binding to the VEGFR-2 receptor. Consequently, serum VEGF is decreased in HIV-positive women, with an increased expression of ICAM-1 and VCAM-1. The presence of the Tat protein in preeclampsia (PE) decreases VEGF. Image adapted from (179).

Tat is rich in arginine and lysine amino acids in a similar sequence to heparin-binding angiogenic factors such as VEGF and basic fibroblast growth factor (bFGF) (180, 181); therefore, it mimics their role by binding and stimulating VEGFR-2 (Flk-1) tyrosine kinase receptor (180, 182, 183) (Figure 5). In addition, due to its similarities to VEGF, Tat mimics VEGF by interfering with the normal development of blood vessels. This lowers VEGF bioavailability, which is required for healthy (234, 236) vessel formation and normal blood flow, potentially leading to PE (184, 185). Other studies have shown reduced VEGF levels in PE (186, 187); this reduction is also linked to the binding of free VEGF to soluble Flt-1 (sFlt-1) (188). sFlt-1 is an antiangiogenic molecule implicated in the pathogenesis of PE (189–191). Even prior to the mother exhibiting clinical symptoms, sFlt-1 levels increase in PE, and these levels are correlated with the severity of PE (192, 193). Tat competes with natural integrin ligands via the Arg–Gly–Asp sequence in the C-terminus and binds to integrins αv β3 and α5 β1 (194). Integrins are receptors for extracellular matrix proteins that are critical for EC adhesion, migration, and invasion (195). Moreover, HIV-1 Tat proteins may induce the expression of inflammatory adhesion regulators ICAM-1 and VCAM-1, signifying a possible mechanism by which HIV-1 infection exacerbates endothelial injury and promotes enhanced atherosclerosis (196–198). These inflammatory processes can also play a role in the nitric oxide regulation, and it can be seen in the processes related to oxidative stress and ADMA build-up.

#### 6.1.3 Nef

The protein Nef stimulates the proliferation of glomerular podocytes through the activation of signal transducer and activator of transcription 3 (Stat3) and mitogen-activated protein kinase (MAPK)-1 and−2 pathways (199). Notably, in the synergy of HIV-associated PE, the hypoxic microenvironment of PE, Nef regulates trophoblast cell invasion and affects angiogenesis via the Stat-3 and MAPK-1 pathways (200). Nef could also promote the inhibition of the ADMA-mediated inhibition of nitric oxide signaling in the vascular endothelium through the activation of inflammatory and oxidative pathways.

### 6.2 Asymmetric dimethylarginine as a therapeutic target in HIV-related preeclampsia

While NO deficiency is characteristic of hypertensive disorders (e.g., PE) and cardiovascular diseases (201), and researchers are seeking ways to reduce ADMA levels, there are instances where NO produced inappropriately or in excessive quantities is cytotoxic, and ADMA may serve a protective role by inhibiting nitric oxide synthase (NOS) activity. The pathological relevance of ADMA extends across multiple disease states, including idiopathic pulmonary hypertension (201), peripheral arterial occlusive disease (202), portal hypertension (203), heart failure (204), hypopituitarism (205), erectile dysfunction (206), aging (201), systemic sclerosis (207), and subarachnoid hemorrhage (208). Elevated ADMA disrupts NO homeostasis by competitively inhibiting eNOS, thereby promoting endothelial dysfunction through impaired vasodilation, heightened oxidative stress, and vascular inflammation (228). Recent evidence has accumulated indicating that consistently elevated levels of ADMA may have a direct positive effect on the management of atherosclerotic vascular diseases, positioning this substance as a promising new target for therapy (209). This potential therapeutic target could theoretically include every phase of the production and removal of ADMA. Therefore, assessing ADMA concentrations can serve as a diagnostic method and may have the potential to be an indirect drug target for various diseases (123, 210). Dysregulation of the ADMA will result in the DDAH and NOS pathway as a significant potential mechanism of endothelial injury in the situation of HIV-associated preeclampsia (220, 221). Chronic immune activation and oxidative stress are linked to HIV infection and may inhibit DDAH activity and thus lowering ADMA metabolism, culminating in the accumulation of circulating ADMA and inhibition of eNOS (221). The subsequent loss of the bioavailability of nitric oxide can be a contributing factor to vascular dysfunction, poor placental perfusion, and the maladaptive endothelial behaviors typical of PE (222).

Angiotensin-converting enzyme inhibitors (ACEIs), angiotensin II type 1 receptor blockers (ARBs), and aldosterone antagonists are widely employed in the management of arterial hypertension and heart failure. Numerous studies have demonstrated that both ACEIs (223, 230) and ARBs are associated with reductions in circulating ADMA levels (119–121, 211); however, the precise mechanisms by which renin-angiotensin-aldosterone system (RAAS) inhibition influences ADMA metabolism remain incompletely understood. Given that DDAH activity is susceptible to inactivation by reactive oxygen species (ROS), ACEIs and ARBs may enhance ADMA clearance through the attenuation of oxidative stress (239). Consistent with this hypothesis, several studies have reported reductions in circulating markers of oxidative stress following treatment with these agents (119–121). Notably, some authors demonstrated that zofenopril, an ACEI possessing sulfhydryl-mediated antioxidant properties, was significantly more effective in lowering ADMA concentrations than enalapril, an ACEI lacking intrinsic antioxidant activity (121).

Moreover, L-arginine was infrequently assessed, but the L-arginine/ADMA ratio may hold greater significance for NOS function than ADMA alone, and arginine levels may be elevated (212) or diminished (213) by pharmacotherapy. Alongside traditional therapy, medicines that particularly target ADMA, such as PRMT inhibitors or DDAH inducers, require more exploration. ADMA may also be addressed with nonpharmacological interventions such as DDAH gene transfer (214), positive airway pressure breathing (215), or weight reduction (122); however, these topics fall outside the purview of this review. Any further research done regarding preeclampsia in relation to HIV should consequently focus on how to implement strategies that can assist in restoring nitric oxide bioavailability by regulating the ADMA–DDAH axis. Such strategies might involve pharmacological therapies that stimulate DDAH functions, mitigate oxidative stress, or elevate the supply of L-arginine and thereby reverse inhibition of eNOS by ADMA and increase endothelial functioning in HIV positive pregnant women. Lastly, the potential utility of agents that increase ADMA (224, 225), such as DDAH inhibitors, should be assessed in the context of unfavorable NO excess caused by septic shock, excitatory neurotoxicity, or tumor angiogenesis. Given that nitric oxide production is linked to the ratio of L-arginine to ADMA, the most effective treatment options for individuals with elevated plasma levels of ADMA include L-arginine replacement therapy and interventions that address the underlying risk factors that lead to increased ADMA levels or decreased L-arginine until a targeted ADMA-reducing therapy is accessible to the public (227). The clinical application of ADMA monitoring or specifically designed interventions in areas with a dense concentration of HIV infection and hypertensive disorders of pregnancy may be logistically and economically problematic, especially in sub-Saharan Africa. However, the discovery of low-cost biomarkers like ADMA or L-arg-ADMA would be useful when it comes to early signs of endothelial dysfunction and a better way to stratify risk in pregnant women living with HIV.

### 6.3 Study limitations

Despite the growing body of research on the involvement of ADMA in PE- and HIV-related vascular dysfunction, several key limitations hinder the current evidence base. Most studies are either observational or cross-sectional, which restricts causal inferences regarding whether increased ADMA directly influences the development of PE or if it is a result of endothelial dysfunction. The sample sizes, particularly in studies involving HIV-positive women with PE, remain limited and geographically specific, which diminishes the applicability of the findings. The lack of defined, pregnancy-specific reference ranges exacerbates the significant variation in ADMA screening methods and sample collection time during gestation, making cross-study comparisons challenging. In addition, many studies fail to account for important confounders such as immunological status (e.g., CD4 count, viral load), ART regimen, and other cardiovascular and metabolic risk factors that may independently affect ADMA levels and preeclampsia risk. At last, translation into clinical practice is limited by the lack of longitudinal and interventional studies assessing whether altering ADMA levels (e.g., by oxidative stress reduction or L-arginine supplementation) can prevent or ameliorate PE, especially in the context of HIV infection (Table 2). In addition, the mechanistic interconnection of HIV proteins, ADMA metabolism and endothelial dysfunction is mostly based on conjectural experiments and observations, thus a prospective mechanistic and interventional study on pregnant populations is warranted, this also indicate the practical obstacles of the application of regular ADMA monitoring or directed therapeutic interventions within restricted healthcare frameworks, especially in areas with a high HIV and preeclampsia infection rate like sub-Saharan Africa.

**Table 2:** Studies showing a link between ADMA, HIV, and preeclampsia.

Study/population	Study design/sample	Key findings	References
ADMA levels preceding clinical PE	Longitudinal study (mid-pregnancy and delivery)	ADMA concentrations were elevated at mid-pregnancy and at delivery in women who developed PE compared with uncomplicated pregnancies.	(59)
ADMA in maternal and fetal circulation	Cross-sectional study; PE and control pregnancies	Elevated maternal ADMA and SDMA levels in preeclampsia; placenta suggested as a potential source.	(127)
ADMA levels in preeclampsia	Case-control study; PE vs. normotensive pregnancies	Maternal serum ADMA concentrations were significantly higher in women with preeclampsia.	(217)
Meta-analysis of ADMA in PE	Meta-analysis of observational studies	Circulating ADMA levels were significantly higher in women with preeclampsia across pooled analyses.	(13)
Maternal serum ADMA in PE vs. controls	Case-control (57 PE vs. 30 normal pregnancies)	Maternal serum ADMA levels were significantly higher in PE vs. normotensive pregnancies, correlating with blood pressure and renal parameters.	(219)
Endothelial impairment in HIV-associated PE	Cross-sectional study; pregnant women stratified by HIV and PE status	ADMA levels were higher in preeclamptic women relative to normotensive controls; HIV status influenced indicators of endothelial dysfunction.	(17, 221)
DDAH gene polymorphisms in PE with HIV	Observational genetic study; 405 pregnant women	SNPs in DDAH1 and DDAH2 genes associated with PE; no direct correlation of polymorphisms with HIV infection status.	(18)

## 7 Conclusions

Vascular disease is closely linked to plasma ADMA levels. In PE, abnormal placental development leads to low oxygen levels, which trigger the release of reactive oxygen species that damage the mother's blood vessel lining. This widespread endothelial impairment occurs because the balance of blood vessel growth is disrupted, resulting in proteinuria and high blood pressure in PE. Asymmetric dimethylarginine, which usually helps maintain a healthy endothelium, becomes imbalanced throughout the body in PE. Higher levels of ADMA, an endogenous competitive inhibitor of endothelial nitric oxide synthase (eNOS), play a role in this process by lowering NO bioavailability and preventing the endothelial-dependent vasodilation (11, 14). Therefore, ADMA–DDAH–NOS signaling pathway problems are becoming more indicated as major causes of endothelial dysfunction in pregnancy hypertensive disorders.

HIV accessory proteins such as gp120, Tat, and Nef cause significant changes in the structure and function of endothelial cells. After HIV infection and ART, higher levels of ROS and oxidative stress often affect the blood vessel lining. Immunosurveillance in response to HIV could also lead to impairment of the DDAH, an enzyme that breaks down ADMA, which subsequently enhances ADMA buildup and subsequent inhibition of nitric oxide production. Recent research that investigated endothelial dysfunction in HIV-related preeclampsia has documented high concentrations of ADMA and a change in the regulation of the vasodilatory pathway, which is in favor of ADMA involvement in the pathomechanism named vascular impairment in this comorbidity. When ART starts, ADMA levels decrease, probably an indication of a lowered systemic inflammatory response and immune activation after viral suppression. The clinical implications of these changes are, however, complicated since certain antiretroviral treatments could be simultaneously directed toward oxidative stress, metabolic imbalances, and endothelial damage. This means that the decrease in ADMA after the initiation of ART does not always result in the prompt amelioration of endothelial functions.

Having both HIV infection and PE worsens endothelial impairment and suggests that ADMA is a strong, though not fully understood, risk factor for PE. HIV-related inflammatory pathway, oxidative stress and dysregulation of the ADMA–DDAH–NOS axis interaction can be an important critical mechanistic connection between the HIV infection and the vascular pathology of PE. The availability of nitric oxide is regulated by the L-arginine/ADMA ratio, which has led to current treatment strategies aimed at restoring this balance through the supplementation of L-arginine and addressing the elements that increase ADMA levels. Any of the above therapeutic interventions that enhance the bioavailability of nitric oxide, increase the activity of DDAH, or suppress oxidative stress can thus form an acceptable approach to ameliorate endothelial dysfunction in HIV-related PE. Developing targeted therapies to reduce ADMA continues to be a significant objective for future applications in clinical settings. Longitudinal and mechanistic experiments are also needed to understand the exact role of ADMA in the overlap between HIV infection and preeclampsia and whether manipulation of the ADMA–DDAH–NOS pathway can have any clinically significant effect in patients with the disease.
